# Treatment of RET-Positive Advanced Medullary Thyroid Cancer with Multi-Tyrosine Kinase Inhibitors—A Retrospective Multi-Center Registry Analysis

**DOI:** 10.3390/cancers14143405

**Published:** 2022-07-13

**Authors:** Viktoria Florentine Koehler, Pia Adam, Carmina Teresa Fuss, Linmiao Jiang, Elke Berg, Karin Frank-Raue, Friedhelm Raue, Eva Hoster, Thomas Knösel, Hans-Ulrich Schildhaus, Thomas Negele, Udo Siebolts, Kerstin Lorenz, Stephanie Allelein, Matthias Schott, Christine Spitzweg, Matthias Kroiss

**Affiliations:** 1 Department of Internal Medicine IV, University Hospital of Munich, LMU Munich, 81377 Munich, Germany; viktoria.koehler@med.uni-muenchen.de (V.F.K.); berg@med.augustinum.de (E.B.); christine.spitzweg@med.uni-muenchen.de (C.S.); 2 Department of Medicine I, Goethe University Hospital, 60590 Frankfurt, Germany; 3 Department of Internal Medicine I, Division of Endocrinology/Diabetology, University of Würzburg, 97080 Würzburg, Germany; adam_p@ukw.de (P.A.); fuss_c@ukw.de (C.T.F.); 4 Institute for Medical Information Processing, Biometry and Epidemiology, LMU Munich, 81377 Munich, Germany; linmiao.jiang@ibe.med.uni-muenchen.de (L.J.); eva.hoster@med.uni-muenchen.de (E.H.); 5 Klinik Augustinum, 81375 Munich, Germany; 6 Private Practice of Endocrinology and Nuclear Medicine, 69120 Heidelberg, Germany; karin.frank.raue@raue-endokrinologie.de (K.F.-R.); friedhelm.raue@raue-endokrinologie.de (F.R.); 7 Department of Pathology, University Hospital of Munich, LMU Munich, 80337 Munich, Germany; thomas.knoesel@med.uni-muenchen.de; 8 Institute of Pathology, University Medical Center Essen, 45147 Essen, Germany; hans-ulrich.schildhaus@uk-essen.de; 9 Department of Surgery, Krankenhaus Martha Maria, 81479 Munich, Germany; thomas.negele@martha-maria.de; 10 Department of Pathology, University Hospital Halle (Saale), Martin-Luther University Halle-Wittenberg, 06108 Halle, Germany; udo.siebolts@uk-halle.de; 11 Department of Visceral, Vascular, and Endocrine Surgery, Martin-Luther University Halle-Wittenberg, 06108 Halle, Germany; kerstin.lorenz@uk-halle.de; 12 Division for Specific Endocrinology, Medical Faculty, University of Düsseldorf, 40225 Düsseldorf, Germany; stephanie.allelein@med.uni-duesseldorf.de (S.A.); matthias.schott@med.uni-duesseldorf.de (M.S.); 13 Division of Endocrinology, Diabetes, Metabolism and Nutrition, Mayo Clinic Rochester, Rochester, MN 55905, USA

**Keywords:** medullary thyroid cancer, rearranged during transfection, variant, multi-tyrosine kinase inhibitor, survival, treatment outcome

## Abstract

**Simple Summary:**

Lately, a more personalized approach in the management of advanced thyroid cancer patients has improved the outcomes, and several novel molecularly guided therapies, including selective RET inhibitors (sRETis), have demonstrated promising efficacy in clinical trials. *RET* (rearranged during transfection) variants are the most prevalent oncogenic event in medullary thyroid cancer (MTC). We here found *RET* oncogene variants in 44/48 prospectively collected MTC tumor samples from patients treated with more unselective kinase inhibitors vandetanib and/or cabozantinib. Our study shows that *RET* variants were highly prevalent in patients with advanced MTC, and the treatment results in *RET*-positive cases were similar to those reported in unselected cohorts.

**Abstract:**

Background: *RET* (rearranged during transfection) variants are the most prevalent oncogenic events in medullary thyroid cancer (MTC). In advanced disease, multi-tyrosine kinase inhibitors (MKIs) cabozantinib and vandetanib are the approved standard treatment irrespective of *RET* status. The actual outcome of patients with *RET*-positive MTC treated with MKIs is ill described. Methods: We here retrospectively determined the *RET* oncogene variant status with a targeted DNA Custom Panel in a prospectively collected cohort of 48 patients with advanced MTC treated with vandetanib and/or cabozantinib at four German referral centers. Progression-free survival (PFS) and overall survival (OS) probabilities were estimated using the Kaplan-Meier method. Results: In total, 44/48 (92%) patients had germline or somatic *RET* variants. The M918T variant was found in 29/44 (66%) cases. In total, 2/32 (6%) patients with a somatic *RET* variant had further somatic variants, while in 1/32 (3%) patient with a germline *RET* variant, additional variants were found. Only 1/48 (2%) patient had a pathogenic *HRAS* variant, and no variants were found in 3 cases. In first-line treatment, the median OS was 53 (95% CI (95% confidence interval), 32–NR (not reached); *n* = 36), and the median PFS was 21 months (12–39; *n* = 33) in *RET*-positive MTC patients. In second-line treatment, the median OS was 18 (13–79; *n* = 22), and the median PFS was 3.5 months (2–14; *n* = 22) in *RET*-positive cases. Conclusions: *RET* variants were highly prevalent in patients with advanced MTC. The treatment results in *RET*-positive cases were similar to those reported in unselected cohorts.

## 1. Introduction

Medullary thyroid cancer (MTC) accounts for 2–5% of all thyroid malignancies and is a tumor arising from the calcitonin-producing parafollicular C cells of the thyroid gland [1]. Hereditary MTC occurs in about 25% of cases as a part of multiple endocrine neoplasia type 2 (MEN2). It is caused by oncogenic germline *RET* (rearranged during transfection) variants. In sporadic MTC, somatic *RET* variants are found in ~65% of cases, among which *RET*M918T is the most frequent somatic variant and is associated with adverse outcomes [2,3,4,5]. Activating variants in the *H-* and *KRAS* genes were found in ~24% of cases using next-generation panel sequencing, while in ~18% of the 181 patients under study, no pathogenic variants were identified [6]. For patients with significant tumor burden, symptoms and/or progressive disease, the multi-tyrosine kinase inhibitors (MKIs) vandetanib and cabozantinib have been approved in the United States and Europe by the United States Food and Drug Administration (FDA) and the European Medicines Agency (EMA), respectively [7]. RET is one among the several targeted kinases of these compounds, and approval is irrespective of the identification of a molecular driver event. While vandetanib inhibits RET tyrosine kinase activity (50% inhibitory concentration (IC_50_) of 130 nM), vascular endothelial growth factor receptor (VEGFR) 2 (IC_50_ of 40 nM) and 3 (IC_50_ of 110 nM) and epidermal growth factor receptor (EGFR) (IC_50_ of 500 nM), cabozantinib targets RET (IC_50_ of 4 nM), VEGFR2 (IC_50_ of 0.035 nM) and hepatocyte growth factor receptor c-Met (IC_50_ of 1.8 nM) [8,9,10,11].

In contrast, highly selective, small-molecule RET kinase inhibitors (sRETis) selpercatinib and pralsetinib have been approved by the FDA for patients with advanced or metastatic MTC in which activating *RET* variants are identified and who require systemic treatment. There is limited evidence of the impacts germline and somatic *RET* oncogene variants may have on the clinical outcome of patients with advanced MTC treated with MKIs such as vandetanib and cabozantinib. For cabozantinib, a post hoc analysis suggested that overall survival (OS) may be significantly longer in patients with the *RET*M918T variant than in patients without this variant or in whom the *RET* status is unknown [12].

To assess the actual clinical course with MKIs specifically in *RET*-positive MTC, we here retrospectively assessed the *RET* variant status in tissue samples from a prospective multi-center registry study at four German tertiary care centers and studied the outcomes specifically after treatment with MKIs in *RET*-positive cases.

## 2. Materials and Methods

### 2.1. Setting

The setting of this registry study was as previously described for an unselected cohort [13]. The study was conducted as part of the German Study Group for Rare Malignant Tumors of the Thyroid and Parathyroid Glands. Prospectively and retrospectively collected data were obtained from records of patients diagnosed with MTC between 1990 and 2019 in four German tertiary care centers. All patients provided written informed consent, and the study was approved by the ethics committee of University of Würzburg (96/13) and subsequently by the ethics committees of all participating centers.

### 2.2. Data Acquisition

**Figure 1:**
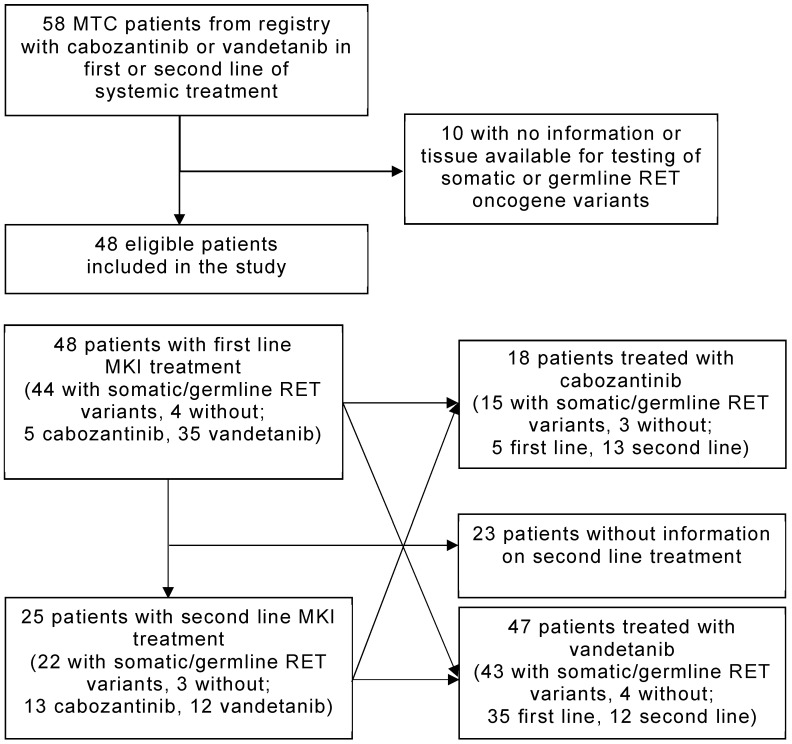
Selection of patients. Abbreviations: MTC, medullary thyroid cancer; RET variant, rearranged-during-transfection variant; MKI, multi-tyrosine kinase inhibitor.

Eligible patients were adults (age ≥ 18 years) with histopathological evidence of MTC with locally advanced disease and/or evidence of distant metastases who underwent MKI treatment with vandetanib and/or cabozantinib [13] with information or tissue available for testing of somatic or germline *RET* oncogene variants. The selection of patients is shown in Figure 1. The primary endpoint of this study was the OS of MTC patients with somatic or germline *RET* oncogene variants during MKI treatment. The secondary endpoints were the assessments of the best objective response rate (based on clinical routine imaging in analogy to RECIST 1.0 and 1.1) and progression-free survival (PFS). Bone metastases were not considered as target lesions, except for the new occurrence of bone metastases upon treatment. As exploratory analyses, we also assessed these end points in patients without *RET* variants.

Time between the first diagnosis and MKI start, Union for International Cancer Control (UICC) stage at the first diagnosis, type of first-line therapy and number of metastases at diagnosis were assessed as potential risk factors affecting the outcome parameters. For OS, patients alive were censored at the cutoff date of 30 October 2020 or at the start of sRETi treatment. Treatment and follow-up of patients were performed according to the local practices of the participating centers. Treatment outcome was assessed locally by imaging (positron emission tomography/computed tomography (PET/CT), CT, magnetic resonance imaging (MRI) of the liver and bone scintigraphy) and measurement of serum calcitonin and carcinoembryonic antigen (CEA) levels every 3–6 months. Clinical data were recorded by trained personnel at all sites.

### 2.3. Sample Selection, DNA/RNA Extraction and Mutation Analysis

Tissue blocks were collected from 43 (90%) patients in Germany with advanced MTC (primary tumor tissue, *n* = 17; tissue of distant metastases, *n* = 11; tissue of lymph node metastases, *n* = 9; unknown, *n* = 6). Five (10%) patients with MEN2 only underwent the analysis of peripheral blood.

Prior to sequencing, specimens were reviewed by an independent pathologist for consistency with the previously established diagnosis. DNA extraction from formalin-fixed, paraffin-embedded (FFPE) tumor tissue, next-generation sequencing (NGS) and bioinformatics were carried out as previously described [14,15].

### 2.4. Statistical Analysis

PFS and OS probabilities were estimated using the Kaplan–Meier method. For exploratory analyses, Cox proportional hazards regression models were used to identify predictive factors affecting the OS of patients with germline/somatic *RET* oncogene variants. Potential predictive factors were selected using backward/stepwise selection based on the p-value (selection threshold of 0.10) or Akaike information criteria (AIC) (−2 × log-likelihood + 2 × degrees of freedom for predictors) and penalized (Lasso) regression methods (10-fold cross-validation). Different selection methods, including backward elimination, stepwise selection and penalized regression, were used to select a consistently robust and reliable set of predictive factors.

The above methods were applied to 4 pooled samples—in patients with first-line MKI treatment, patients with second-line MKI treatment, patients treated with vandetanib and patients treated with cabozantinib. Due to the limited sample size, no statistical tests comparing the characteristics or outcomes of patients with and without germline/somatic *RET* variants were performed. Statistical analyses were performed with R Statistical Software Version 4.0.4 (R Foundation for Statistical Computing, Vienna, Austria). Microsoft Office Excel Version 16.55 was used for additional analyses.

## 3. Results

### 3.1. Clinical Characteristics

At four German tertiary care centers, 48 patients (36 males and 12 females) with locally advanced MTC and/or evidence of distant metastases undergoing MKI treatment were included. Table 1 shows the baseline clinical characteristics of the study population. The median follow-up from the first MTC diagnosis was 6 years (range of 0–30). Most patients presented with a germline or somatic *RET* variant (44; 92%). The median age at the initial diagnosis of sporadic MTC was 47 years (range of 23–78), and it was 42 years (range of 17–61) for patients with hereditary MTC. At the diagnosis of metastatic disease, the median age was 50 years, and tumors were already metastatic at diagnosis in 30 (63%) patients. Before MKI initiation, the median calcitonin doubling time (CDT) was 8 months (range of 4–31). The median age at MKI initiation was 55 years (range of 22–79), and the median time between initial diagnosis and MKI start was 37 months (range of 0–242). In patients with distant metastases at initial diagnosis, the median time between initial diagnosis and MKI start was 18 months (range of 0–199). The median starting doses of vandetanib and cabozantinib were 300 mg/day (range of 100–300) and 80 mg/day (range of 60–140), respectively. A total of 27 (63%) patients started vandetanib treatment at the approved dosage of 300 mg/day, and 4 (27%) patients started cabozantinib at the approved dosage of 140 mg/day.

### 3.2. Analysis of Genetic Alterations Occurring in MTC Cases

In total, 54 genetic alterations were detected in 48 MTC cases. The genetic features of the study are summarized in Table A1. Pathogenic variants in the *RET* proto-oncogene were found in 44/48 (92%) cases. In total, 7/44 (16%) *RET* variants were germline, and 32/44 (73%) were somatic, while in 5/44 (11%) patients with tumoral *RET* variant, blood was not available for comparison. However, there was no clinical evidence for MEN2 in patients without analyses of peripheral blood. The *RET* variant was the M918T variant in 29/44 (66%) cases. Codon 634 was altered in 4/44 (9%) cases with different amino acid substitutions. In total, 2/44 (5%) cases showed a codon 620 variant, 2/44 (5%) an A833F variant, 2/44 (5%) an exon 11 variant, 1/44 (2%) case a *RET* rearrangement and 1/44 (2%) c.2694_2705del + p.D898_E901del, and 1/44 (2%) case showed a C618R variant. Details about the *RET* variant status were not obtainable in two cases who did not undergo panel sequencing. In total, 2/32 (6%) patients with a somatic *RET* variant had further somatic variants in the *KRAS*, *TP53* and *HER2* genes. In 1/7 (14%) patient with a germline *RET* variant, additional variants in the *EGFR* gene, *CDKN2B* gene and the *MAP2K1* gene were found. In total, 3/4 patients without a *RET* variant showed no detectable variants, and one patient had the pathogenic *HRAS* variant (exon 3 c.181C>A (p.Gln61Lys)).

### 3.3. Multi-Tyrosine Kinase Inhibitor Therapy

The treatment and patient characteristics are shown in Table 1. The median follow-up from the start of MKI treatment was 34 months (range of 3–164). Vandetanib and/or cabozantinib were administered in all 48 patients. Eight (17%) patients received a different MKI as first-line treatment (sorafenib or imatinib prior to approval of vandetanib). A total of 20 patients (42%) received two MKIs, while 6 (13%) patients received three MKIs, and 2 (4%) patients received five MKIs.

### 3.4. Outcome after First- and Second-Line Treatments

The characteristics of first-line treatment with cabozantinib/vandetanib are summarized in Table A2. The outcome data are summarized in Table A3.

A total of 35 (73%) patients received vandetanib as first-line treatment, 33 patients with a *RET* variant and 2 patients without a *RET* variant. Five patients (10%) received cabozantinib as first-line treatment, three patients with a *RET* variant and two patients without a *RET* variant.

In the first-line treatment of patients with a *RET* variant, the median OS was 53 months (95% CI, 32–NR), and the median PFS was 21 months (95% CI, 12–39).

A total of 12 (48%) patients received vandetanib as second-line treatment, 10 patients with a *RET* variant and 2 patients without a *RET* variant. A total of 13 patients (52%) received cabozantinib as second-line treatment, 12 patients with a *RET* variant and 1 patient without a *RET* variant.

In the second-line treatment of patients with a *RET* variant, the median OS was 18 (95% CI, 13–79), and the median PFS was 3.5 months (95% CI, 2–14).

In the very small group of patients without a *RET* variant (n = 4), the median OS and PFS for first-line treatment were 20 months (95% CI, 5–NR) and 4 months (95% CI, 0–NR), respectively. For second-line treatment, the median OS and PFS were 14 (95% CI, 5–NR) and 3 months (95% Cl, 3–NR), respectively.

### 3.5. Outcome with Vandetanib and Cabozantinib Treatments

When analyzed irrespectively of treatment line, 18 patients received cabozantinib and 47 vandetanib. The outcome data are summarized in Table A3. For patients with a *RET* variant (vandetanib *n* = 43; cabozantinib *n* = 15), the median OS for vandetanib was 54 (95% CI, 36–NR), and the median PFS was 18 months (95% CI, 12–33). For cabozantinib treatment, the median OS was 14 (95% CI, 10–NR), and the median PFS was 2 months (95% CI, 1–14).

For patients without a *RET* variant (vandetanib *n* = 4; cabozantinib *n* = 3), the median OS and PFS for vandetanib were 10 months (95% CI, 5–NR) and 2 months (95% Cl, 0–NR), while for cabozantinib, they were 22 months (95% CI, 20–NR) and 10 months (95% Cl, 8–NR)

### 3.6. Biochemical Response of First-Line Treatment

Calcitonin as well as CEA (calcitonin *n* = 24; CEA *n* = 22) showed a significant reduction in patients with a *RET* variant (77% reduction, *p* < 0.001 ***; 74% reduction, *p* = 0.007 **); patients without a *RET* variant (calcitonin *n* = 3, CEA *n* = 1) showed no significant reductions in calcitonin (*p* = 0.5). The data on CEA were not sufficient for analysis.

### 3.7. Predictive Factors Affecting OS in First- and Second-Line Treatments

The associations of predictive factors with OS are summarized in Table 2. Patients with a longer time interval between the first diagnosis and MKI initiation and patients with a higher number of metastases at the first diagnosis experienced a longer OS.

Patients with *RET* variants receiving second-line treatment had a longer OS when the time interval between the first diagnosis and MKI initiation was longer and a lower number of metastases was present at the first diagnosis, suggesting less aggressive disease course, and when treated in second line with vandetanib.

### 3.8. Safety and Tolerability

In 9 (21%) patients taking vandetanib and 9 (60%) patients taking cabozantinib, treatment-emergent adverse events (TEAEs) were the reason for treatment discontinuation. The TEAEs of vandetanib and cabozantinib in patients with a *RET* variant are summarized in Table 3. In vandetanib-treated patients, the most frequently reported TEAEs were diarrhea (53%), skin rash (44%) and fatigue (28%); in cabozantinib-treated patients, they were loss of appetite/loss of weight (53%), diarrhea (40%) and fatigue (40%). Patients taking cabozantinib showed a higher incidence of laboratory abnormalities, including blood count changes and thyroid-stimulating hormone (TSH) elevation, than vandetanib-treated patients. In vandetanib-treated patients, 10 (23%) patients showed a prolongation of the QT interval, while no patients taking cabozantinib had a documented prolongation of the QT interval. Furthermore, hand–foot syndrome was more often noted in patients taking cabozantinib than in those taking vandetanib (5 (33%) vs. 1 (2%)).

## 4. Discussion

To our knowledge, this is the first analysis describing the response to approved MKIs vandetanib and cabozantinib specifically in patients with a somatic or germline *RET* variant outside of a clinical trial.

In our series, 92% of cases harbored *RET* variants, confirming that *RET*, particularly the M918T variant, is the main driver oncogene in advanced MTC. The prevalence of *RET*-negative cases was 8%, and the proportion of cases in which no specific driver events were found was only 6%. Compared with previous data [4,6], as well as data from the phase 3 EXAM trial [12], *RET*-positive cases were more frequent in our series. At the same time, there was a remarkably small proportion of cases with no driver events. These differences are most likely due to the selection of patients, as we only included patients with advanced disease undergoing treatment with vandetanib and/or cabozantinib, as well as differences in sequencing technologies. The high prevalence of *RET* variants in our cohort provides a rationale for treatment with sRETis in the majority of patients.

Limitations of our study include the potential selection bias towards *RET*-positive MTC due to the participation of some centers in clinical trials of sRETis. Furthermore, the small group size of *RET*-negative patients precluded comparisons between *RET*-altered and *RET*-negative cases and the analysis of individual MKIs according to the treatment line. Missing data and differences in response assessment and follow-up are due to the—in part—retrospective nature of a registry study.

The outcome after vandetanib treatment in *RET*-positive patients, regardless of the treatment line, was consistent with previously reported results from our unselected cohort of 48 patients with advanced MTC undergoing MKI treatment with vandetanib and/or cabozantinib, which showed a median OS of 53 months and a median PFS of 17 months [13]. Thirty-four *RET*-positive patients were also part of that unselected series.

The median OS of 14 months and PFS of 2 months in cabozantinib-treated *RET*-positive patients, regardless of the treatment line, were lower than it was in our previous study [13]. We postulate that the lower response rates of cabozantinib-treated patients were most likely due to the fact that the results included a higher rate of patients receiving cabozantinib as second-line treatment after PD in first-line treatment than previously reported results (1:4 vs. 1:2 in our prior study). Only 27% of patients received the full dosage of 140 mg/day at treatment initiation (30% in our unselected series), and 67% needed a dose reduction, compared to 61% in our prior study [13].

According to the multiple Cox regression analysis, there were no signals of superiority for a particular MKI regimen in the first-line setting with respect to OS. Patients undergoing second-line vandetanib treatment showed a longer OS than patients receiving cabozantinib. Nevertheless, these data need to be interpreted with caution due to the limited number of patients receiving first-line treatment with cabozantinib and the limited number of patients receiving second-line treatment with vandetanib after first-line treatment with cabozantinib (8/10 patients received vandetanib after first-line treatment with sorafenib or imatinib).

The safety and tolerability, including the rate of treatment discontinuation due to TEAEs of vandetanib and cabozantinib, were comparable with the results of our prior study [13].

In an indirect comparison with the phase 1/2 trial of selpercatinib in patients with *RET*-positive MTC showing an ORR (objective response rate) of 69% and 72% in previously treated and not-treated patients by independent review, respectively, the ORR in our cohort ranged between 27% and 32% depending on the treatment line and compound [16]. The phase 1/2 open-label study of pralsetinib including patients with *RET*-positive MTC also showed a higher ORR of 60% in pre-treated patients and 66% in patients who did not receive prior vandetanib or cabozantinib [17]. The small proportion of *RET-*negative cases in our series is similar to that in the phase 3 ZETA trial, where only 2/231 (1%) of patients in the vandetanib group and 6/100 (6%) of patients in the placebo group with MTC were *RET* negative [2]. Furthermore, a substantial percentage of patients were *RET* unknown; therefore, the subgroup analyses of PFS and ORR by *RET* variant status were inconclusive [2]. Taking data from the amplification-refractory mutation system (ARMS) assay into account, which specifically detects the most common *RET* variant, M918T, patients with sporadic MTC showed benefit from vandetanib treatment whether their tumors were *RET*M918T positive or negative; however, the response rate was greater in those who had the *RET*M918T variant [2].

In an exploratory assessment of OS, PFS and ORR in the EXAM trial, cabozantinib appeared to be more active in patients who were *RET*M918T positive than in those who were *RET*M918T negative [12]. The median OS was 44 months for patients receiving cabozantinib versus 19 months for placebo, with corresponding values of 20 versus 22 months in the *RET*M918T-negative subgroup [12]. Interestingly, the difference in the OS of *RET*M918T-negative patients receiving cabozantinib versus placebo was only <3 months.

Even if the group of *RET*-negative cases was small, the particularly short PFS and OS appear to contradict the notion that *RET*M918T is associated with aggressive disease and poor prognosis [4,18]. In the series by Elisei et al., patients with an *RET* variant showed a lower survival rate in a long-term follow-up and the highest probability to have persistence of the disease [4]. Furthermore, Schilling et al. showed a more aggressive development of distant metastases during follow-up with decreased metastasis-free survival and a significantly lower survival rate in patients with the *RET*M918T variant than that in patients with a wild-type sequence in that codon [18]. This may reflect the differences between patient groups at different stages of the disease course and the impact of RET-directed treatment. This is in line with data showing *RET* variants to be significantly less frequent in small-sized tumors and much more frequent in advanced metastatic cases [19,20].

## 5. Conclusions

In conclusion, our study shows that patients with advanced MTC receiving MKI treatment had a high prevalence of *RET* variants. The treatment results in *RET*-positive cases were similar to those reported in unselected cohorts, providing a rationale for treatment with sRETis in the majority of patients.

## Figures and Tables

**Table 1:** Patient characteristics of the study cohort.

Characteristics Prior to MKI	With *RET* Variant	Without *RET* Variant
Number of patients	44	4
Germline *RET* variant	7 (16%)	0
Somatic *RET* variant (no germline variant)	32 (73%)	0
Somatic *RET* variant (unknown germline variant)	5 (11%)	0
Male sex	33 (75%)	3 (75%)
Median age at the first diagnosis (years) (range)	45 (15–74)	62 (56–78)
UICC stage at the first diagnosis		
III	6 (15%; *n* = 41)	0
IV	35 (85%; *n* = 41)	3 (100%; *n* = 3)
Lymph node metastases at the first diagnosis	42 (100%; *n* = 42)	3 (75%)
Distant metastases at the first diagnosis	26 (72%; *n* = 36)	3 (100%; *n* = 3)
Brain	0	0
Lung	16 (62%)	1 (33%)
Liver	10 (38%)	2 (67%)
Mediastinum	11 (44%; *n* = 25)	0
Bone	12 (46%)	2 (67%)
Initial thyroidectomy	41 (93%)	3 (75%)
Surgery for metastases	11 (26%; *n* = 43)	0
Calcitonin doubling time prior to MKI start (months) median (range)	8 (4–31; *n* = 18)	NA
Peptide receptor radionuclide therapy prior to MKI	5 (12%; *n* = 43)	0
Chemotherapy prior to MKI	1 (2%)	0
Local radiation therapy prior to MKI	6 (14%)	0
Radiation therapy of metastatic sites prior to MKI	8 (18%)	0
Ablative procedures prior to MKI	3 (7%)	0
**Characteristics at MKI Initiation**		
Indication for MKI therapy		
Extensively metastatic disease at diagnosis	15 (35%; *n* = 43)	4 (100%)
Morphological progression	28 (65%; *n* = 43)	0
Median age at MKI initiation (range)	53 (22–79)	62 (56–78)
Median months between the first diagnosis and MKI initiation (range)	48 (0–242)	2 (2–7)
Lymph node metastases at MKI initiation	41 (93%)	3 (100%; *n* = 3)
Distant metastases at MKI initiation	42 (98%; *n* = 43)	4 (100%)
Brain	2 (5%)	0
Lung	23 (55%)	1 (25%)
Liver	23 (55%)	3 (75%)
Mediastinum	20 (48%)	0
Bone	23 (55%)	3 (75%)
Median calcitonin level (pg/mL) at MKI initiation (range)	2220 (4–254,000; *n* = 38)	600 (396–22,224; *n* = 3)
Median CEA level (ng/mL) at MKI initiation (range)	137 (3–3360; *n* = 35)	31.4 (*n* = 1)
First-line therapy		
Cabozantinib	3 (7%)	2 (50%)
Vandetanib	33 (75%)	2 (50%)
Sorafenib	7 (16%)	0
Imatinib	1 (2%)	0

Abbreviations: MKI, multi-tyrosine kinase inhibitor; RET variant, rearranged-during-transfection variant; UICC, Union for International Cancer Control; CEA, carcinoembryonic antigen.

**Table 2:** Associations of different baseline features with OS in first-line and second-line treatment.

Multiple Cox Regression	
	Predictive Factors (Coefficients) (Candidate Predictive Factors: Months between the First Diagnosis and MKI Start (Numeric), UICC Stage at the First Diagnosis (Factor—III, IV), Type of First-Line Therapy (Factor—Cabozantinib, Vandetanib), Number of Metastases at Diagnosis (Numeric))
*p*-value-based backward model selection (threshold of 0.10)	Number of metastases at diagnosis (−0.558)
*p*-value-based stepwise model selection (threshold of 0.10)	Number of metastases at diagnosis (−0.558)
AIC-based backward model selection	Months between the first diagnosis and MKI start (−0.008) Number of metastases at diagnosis (−0.652)
AIC-based stepwise model selection	Months between the first diagnosis and MKI start (−0.008) Number of metastases at diagnosis (−0.652)
Penalized regression (Lasso)-based model selection (*λ* = 0.08)	Months between the first diagnosis and MKI start (−0.003) Number of metastases at diagnosis (−0.401)
**Multiple Cox Regression**	
	**Predictive Factors (Coefficients) (Candidate Predictive Factors: Months Between the First Diagnosis and Second-Line MKI Start, UICC Stage at the First Diagnosis, Type of Second-Line Therapy, Number of Metastases at Diagnosis)**
*p*-value-based backward model selection (threshold of 0.10)	Months between the first diagnosis and second-line MKI start (−0.008) Type of second-line therapy (vandetanib vs. cabozantinib, −1.607)
*p*-value-based stepwise model selection (threshold of 0.10)	Type of second-line therapy (vandetanib vs. cabozantinib, −1.505)
AIC-based backward model selection	Months between the first diagnosis and second-line MKI start (−0.008) Type of second-line therapy (vandetanib vs. cabozantinib, −1.607)
AIC-based stepwise model selection	Months between the first diagnosis and second-line MKI start (−0.008) Type of second-line therapy (vandetanib vs. cabozantinib, −1.607)
Penalized regression (Lasso)-based model selection (*λ* = 0.22)	Months between the first diagnosis and second-line MKI start (−0.003) Type of second-line therapy (vandetanib vs. cabozantinib, −0.731) Number of metastases at diagnosis (0.065)

Abbreviations: AIC, Akaike information criterium; MKI, multi-tyrosine kinase inhibitor; UICC, Union for International Cancer Control.

**Table 3:** TEAEs in patients with *RET*-positive MTC treated with vandetanib and/or cabozantinib.

	Cabozantinib (*n* = 15)	Vandetanib (*n* = 43)
Discontinuation of/change in therapy due to drug intolerance	9 (60%)	9 (21%)
Bleeding	0	3 (7%)
Change in blood count	2 (13%)	3 (7%)
Electrolyte change	1 (7%)	4 (9%)
Mucositis	5 (33%)	3 (7%)
Diarrhea	6 (40%)	23 (53%)
Dysgeusia/Ageusia	1 (7%)	0
Fatigue	6 (40%)	12 (28%)
Fistula formation	0	1 (2%)
Hand–foot syndrome	5 (33%)	1 (2%; *n* = 42)
Hypertension	0	3 (7%)
Infection	3 (20%)	1 (2%)
Decreased appetite/weight loss	8 (53%)	9 (21%)
Nausea	2 (13%)	3 (7%; *n* = 42)
QTc interval prolongation	0	10 (23%)
Proteinuria	1 (7%)	0
Skin rash	2 (13%)	19 (44%)
TSH elevation	2 (14%; *n* = 14)	0
Thrombosis/thromboembolism	0	1 (2%; *n* = 42)
Vomiting	1 (7%)	2 (5%)
Loss of kidney function	1 (7%)	3 (7%)
Need for dose reduction	10 (67%)	14 (33%)

Abbreviations: TEAEs, treatment-emergent adverse events; TSH, Thyroid-stimulating hormone.

## Data Availability

Not applicable.

## Appendix A

**Table A1:** Genetic features of the study cohort.

Patient No.	*RET* Mutation; Codon	Exon	Biological Significance	Gene; Codon of Further Mutations	Biological Significance
**Hereditary MTC**					
1	yes; M918T	16	pathogenic		
2	yes; M918T	16	pathogenic	unknown	
3	yes; C634R	11	pathogenic	EGFR-V843I; CDKN2B-T95M; MAP2K1-D67N	likely pathogenic; VUS; pathogenic
4	yes; C634R	11	pathogenic		
5	yes; C620G	10	pathogenic	unknown	
6	yes; C620S	10	pathogenic		
7	yes; unknown			unknown	
**Sporadic MTC**					
8	yes; M918T	16	pathogenic	ERBB2-D1115V	VUS
9	yes; M918T	16	pathogenic	KRAS-G12V; PIK3CA-E542V; TP53-A39fs	pathogenic; pathogenic; pathogenic
10	yes; M918T	16	pathogenic		
11	yes; M918T	16	pathogenic		
12	yes; M918T	16	pathogenic		
13	yes; M918T	16	pathogenic		
14	yes; M918T	16	pathogenic		
15	yes; M918T	16	pathogenic		
16	yes; M918T	16	pathogenic		
17	yes; M918T	16	pathogenic		
18	yes; M918T	16	pathogenic		
19	yes; M918T	16	pathogenic		
20	yes; M918T	16	pathogenic		
21	yes; M918T	16	pathogenic		
22	yes; M918T	16	pathogenic		
23	yes; M918T	16	pathogenic		
24	yes; M918T	16	pathogenic		
25	yes; M918T	16	pathogenic		
26	yes; M918T	16	pathogenic		
27	yes; M918T	16	pathogenic		
28	yes; M918T	16	pathogenic		
29	yes; M918T	16	pathogenic		
30	yes; A883F	15	pathogenic		
31	yes; A883F	15	pathogenic		
32	yes; C618R	10	pathogenic		
33	yes; C634R	11	pathogenic		
34	yes; p.Glu632_Cys634delinsGly	11	pathogenic		
35	yes; A886G	11	pathogenic		
36	yes; 30 bp insertion; c.1936_1937ins30—p.Ser645_ Phe646insCysAlaArgAlaAlaAlaValLeuPheSer	11	VUS		
37	yes; p.D898_E901del		pathogenic		
38	yes; 5′/3′-imbalance		VUS		
39	yes; unknown				
40	no			HRASQ61L	pathogenic
41	no				
42	no				
**Unknown Germline Status**					
43	yes; M918T	16			
44	yes; M918T	16			
45	yes; M918T	16			
46	yes; M918T	16			
47	yes; M918T	16			
48	no				

Abbreviations: MTC, medullary thyroid cancer; RET variant, rearranged-during-transfection variant; VUS, variant of uncertain significance.

**Table A2:** Characteristics of patients with cabozaninib/vandetanib as first-line treatment.

Characteristics Prior to MKI	With *RET* Variant	Without *RET* Variant
Number of patients	36	4
Germline *RET* variant	7 (19%)	0
Somatic *RET* variant (no germline variant)	26 (72%)	0
Somatic *RET* variant (unknown germline variant)	3 (8%)	0
Male sex	29 (81%)	3 (75%)
Median age at the first diagnosis (years) (range)	46 (15–74)	62 (56–78)
UICC stage at the first diagnosis		
III	6 (18%; *n* = 33)	0
IV	27 (82%; *n* = 33)	3 (100%; *n* = 3)
Lymphatic metastases at the first diagnosis	34 (100%; *n* = 34)	3 (75%)
Distant metastases at the first diagnosis	23 (77%; *n* = 30)	3 (100%; *n* = 3)
Brain	0	0
Lung	14 (61%)	1 (33%)
Liver	9 (39%)	2 (67%)
Mediastinum	9 (41%; *n* = 22)	0
Bone	11 (48%)	2 (67%)
Initial thyroidectomy	33 (92%)	3 (75%)
Surgery for metastases	7 (20%; *n* = 35)	0
Calcitonin doubling time prior to MKI start (months) median (range)	8 (4–31; *n* = 15)	NA
Peptide receptor radionuclide therapy prior to MKI	3 (9%; *n* = 35)	0
Chemotherapy prior to MKI	1 (3%)	0
Local radiation therapy prior to MKI	5 (14%)	0
Radiation therapy of metastatic sites prior to MKI	8 (22%)	0
Ablative procedures prior to MKI	3 (8%)	0
**Characteristics at MKI Initiation**		
Indication for MKI therapy		
Extensive metastases at the first diagnosis	13 (37; *n* = 35)	4 (100%)
Morphological progression	22 (63%; *n* = 35)	0
Median age at MKI initiation (range)	56 (22–79)	62 (56–78)
Months between the first diagnosis and MKI initiation median (range)	36 (0–242)	2 (2–7)
Lymphatic metastases at MKI initiation	33 (92%)	3 (100%; *n* = 3)
Distant metastases at MKI initiation	34 (97%; *n* = 35)	4 (100%)
Brain	1 (3%)	0
Lung	17 (50%)	1 (25%)
Liver	21 (62%)	3 (75%)
Mediastinum	16 (47%)	0
Bone	19 (56%)	3 (75%)
Median calcitonin level (pg/mL) at MKI initiation (range)	1863 (4–89,300; *n* = 31)	600 (396–22,224; *n* = 3)
Median CEA level (ng/mL) at MKI initiation (range)	137 (3–3360; *n* = 28)	31.4 (*n* = 1)
First-line therapy		
Cabozantinib	3 (8%)	2 (50%)
Vandetanib	33 (92%)	2 (50%)

Abbreviations: MKI, multi-tyrosine kinase inhibitor; RET variant, rearranged-during-transfection variant; UICC, Union for International Cancer Control; NA, not available; CEA, carcinoembryonic antigen.

**Table A3:** MKI treatment response rates of first-line treatment, second-line treatment, vandetanib-treated patients and cabozantinib-treated patients.

First-Line Treatment	With *RET* Variant (*n* = 36)	Without *RET* Variant (*n* = 4)
Median duration of first-line treatment (months) (range)	21 (1–149; *n* = 36)	7 (3–14)
Best response		
PD	5 (15%; *n* = 34)	3 (75%)
SD 8–24 weeks	3 (9%; *n* = 34)	0
SD ≥ 24 weeks	15 (44%; *n* = 34)	1 (25%)
PR	11 (32%; *n* = 34)	0
CR	0	0
Time interval from start of first-line therapy to the start of second-line therapy (months) (range)	16 (0–84; *n* = 14)	9 (4–15; *n* = 3)
Discontinuation of therapy	27 (75%)	4 (100%)
PD	18 (67%)	2 (50%)
Drug intolerance	9 (33%)	2 (50%)
**Second-line treatment**	**With *RET* variant ** **(*n* = 22)**	**Without *RET* variant ** **(*n* = 3)**
Median duration of second-line treatment (months) (range)	10 (1–100; *n* = 21)	8 (3–13; *n* = 2)
Best response		
PD	3 (19%)	1 (33%)
SD 8–24 weeks	8 (36%)	1 (33%)
SD ≥ 24 weeks	4 (18%)	1 (33%)
PR	6 (27%)	0
CR	0	0
Discontinuation of therapy	20 (91%)	2 (67%)
PD	11 (55%)	2 (100%)
Drug intolerance	9 (45%)	0
**Vandetanib-treated patients**	**With *RET* Variant ** **(*n* = 43)**	**Without *RET* Variant ** **(*n* = 4)**
Median duration of vandetanib treatment (months) (range)	20 (1–149; *n* = 42)	3 (3–4; *n* = 3)
Best response		
PD	4 (10%; *n* = 41)	3 (75%)
SD 8–24 weeks	6 (15%; *n* = 41)	1 (25%)
SD ≥ 24 weeks	18 (44%; *n* = 41)	0
PR	13 (32%; *n* = 41)	0
CR	0	0
Discontinuation of therapy	33 (77%)	4 (100%)
Progression	24 (73%)	3 (75%)
Drug intolerance	9 (27%)	1 (25%)
**Cabozantinib-treated patients**	**With *RET* Variant ** **(*n* = 15)**	**Without *RET* Variant ** **(*n* = 3)**
Median duration of cabozantinib treatment (months) (range)	7 (1–39)	13 (9–14)
Best response		
PD	4 (27%)	1 (33%)
SD 8–24 weeks	5 (33%)	0
SD ≥ 24 weeks	1 (7%)	2 (67%)
PR	4 (27%)	0
CR	0	0
Discontinuation of therapy	14 (93%)	2 (67%)
Progression	5 (36%)	1 (50%)
Drug intolerance	9 (64%)	1 (50%)

Abbreviations: MKI, multi-tyrosine kinase inhibitor; RET variant, rearranged-during-transfection variant; PD, progressive disease; SD, stable disease; PR, partial response; CR, complete response.
